# Effects of Roux-en-Y Gastric Bypass on Fasting and Postprandial Levels of the Inflammatory Markers YKL-40 and MCP-1 in Patients with Type 2 Diabetes and Glucose Tolerant Subjects

**DOI:** 10.1155/2013/361781

**Published:** 2013-11-03

**Authors:** Stine Brinkløv Thomsen, Camilla Noelle Rathcke, Nils Bruun Jørgensen, Sten Madsbad, Henrik Vestergaard

**Affiliations:** ^1^Novo Nordisk Foundation Center for Basic Metabolic Research, Faculty of Health and Medical Sciences, University of Copenhagen, Universitetsparken 1, 2100 Copenhagen, Denmark; ^2^Department of Medicine, Center of Endocrinology and Metabolism, Copenhagen University Hospital Herlev, Herlev Ringvej 75, 2730 Herlev, Denmark; ^3^Department of Internal Medicine, Amager Hospital, Italiensvej 1, 2300 Copenhagen, Denmark; ^4^Department of Biomedical Sciences, Faculty of Health and Medical Sciences, University of Copenhagen, Universitetsparken 1, 2100 Copenhagen, Denmark; ^5^Department of Endocrinology, Hvidovre Hospital, Kettegård Alle 30, 2650 Hvidovre, Denmark

## Abstract

*Background*. The inflammatory markers YKL-40 and monocyte chemoattractant protein-1 (MCP-1) are elevated in morbidly obese patients and decline after weight loss. The objective of our study was to investigate the possible changes of YKL-40 and MCP-1, in both the fasting and the postprandial states, following Roux-en-Y gastric bypass (RYGB) in subjects with type 2 diabetes (T2D) and normal glucose tolerance (NGT). *Methods*. Ten obese patients with T2D and 10 subjects with NGT were examined in the fasting state and after a standard meal prior to and after (1 week, 3 months, and 1 year) RYGB. *Results*. Fasting state MCP-1 levels decreased after RYGB in both groups (*P* values < 0.0001) whereas fasting YKL-40 levels were unchanged (*P* values ≥ 0.120). Postprandial MCP-1 levels showed a tendency towards a decrease on most study days; however, the changes were only significant at 1 week (*P* = 0.001) and 1 yr (*P* < 0.0001) in the T2D group and at 3 mo after RYGB in the NGT group (*P* = 0.009). YKL-40 levels showed a slight, postprandial suppression on all study days in the T2D group (all *P* values ≤ 0.021). *Conclusions*. Fasting MCP-1 levels, but not YKL-40 levels, decrease after RYGB in subjects with T2D and NGT. Postprandial changes of inflammatory markers are discrete and inconsistent.

## 1. Introduction

Obesity is an increasing problem worldwide, and especially visceral obesity is strongly linked to the risk of type 2 diabetes and cardiovascular disease (CVD) [1–3]. All of these conditions are characterized by a state of low-grade inflammation with elevated levels of proinflammatory cytokines and acute-phase reactants [4, 5]. It has previously been demonstrated that the intake of a high-fat diet causes elevated levels of bacterial lipopolysaccharide (LPS) in humans [6], which, in turn, trigger the secretion of pro-inflammatory cytokines [7]. Moreover, continuously elevated levels of LPS lead to body weight gain and diabetes in mouse models, possibly through the activation of the LPS receptor CD14 and the subsequent modulation of metabolism [8].

The glycoprotein YKL-40, also named chitinase-3-like-1 (*CHI3L1*), is an inflammatory marker, found to be elevated in conditions characterized by low-grade inflammation, such as myocardial infarction [9, 10], and stable coronary artery disease [11], type 2 diabetes [12, 13], and morbid obesity [14, 15]. In patients with stable coronary artery disease, YKL-40 is correlated positively with triglyceride [16], and in type 2 diabetes patients, YKL-40 correlates positively with triglyceride and free fatty acid (FFA) levels [13]. 

Monocyte chemoattractant protein-1 (MCP-1 or CCL-2) is a member of the CC chemokine subfamily, known to recruit cells of the immune system to the peripheral tissues during infection, injury, and other inflammatory conditions [17]. MCP-1 is thought to play a role in the development of inflammation and insulin resistance (IR) in obese mouse models [18] and may be important to the atherogenic process [19]. A strong association between MCP-1 and other inflammatory mediators has previously been demonstrated [14, 15]. 

Roux-en-Y gastric bypass (RYGB) leads to a mean weight loss of more than 50% of the excess body weight [20]. Furthermore, RYGB is more effective in treating type 2 diabetes than conventional medical therapy, and in many patients, improvement in glucose metabolism occurs just days after surgery, before a major weight loss has occurred [21, 22]. These early changes in glucose metabolism are associated with improved hepatic IR and improved beta-cell function, while peripheral IR improves as patients lose weight [23–25]. To our knowledge, previous studies have focused solely on the effect of RYGB on fasting levels of inflammatory markers, and levels of several inflammatory mediators, including C-reactive protein [26], MCP-1 [27, 28], and YKL-40 [14], have been shown to decrease after weight loss. Monte et al. recently documented that RYGB reduces the fasting plasma levels of LPS and other pro-inflammatory mediators in type 2 diabetes, and it is plausible that this reduction in the inflammatory state is responsible, at least in part, for the improvement of the glucose metabolism [27]. However, the decrease of inflammatory markers observed after RYGB-induced weight loss could also originate from the natural discontinuation of an excessive food intake caused by the operation [27], and thus it would be interesting to observe the postprandial effect on the inflammatory response after bariatric surgery.

In this study, therefore, we investigated the changes in serum levels of the inflammatory markers YKL-40 and MCP-1 in the fasting state and during mixed meal tests in a group of obese patients with type 2 diabetes and a group of obese subjects with normal glucose tolerance (NGT), previous to and after (1 week (wk), 3 months (mo), and 1 year (yr)) RYGB. We hypothesized that the presurgery “inflammatory load” would differ between the two study groups, with higher levels of inflammatory markers in the type 2 diabetes group, and that these levels would decrease in both groups after RYGB. We speculate if the possible differences between the two groups would eventually equalize as an expression of a “normalisation” of the inflammatory state, or if the type 2 diabetes group would permanently have a higher degree of inflammation. Furthermore, we wished to investigate whether the mixed meal tests would induce a temporary inflammatory response with increasing levels of the inflammatory markers, and whether this response would attenuate after RYGB. This study could add to the current knowledge of low-grade inflammation in obesity and type 2 diabetes and of the changes in weight and metabolism caused by bariatric surgery, and, furthermore, it will be a basis for further studies regarding the impact of bariatric surgery on the possible postprandial inflammatory response measured by YKL-40 and MCP-1. This can ultimately be of great importance to clinicians selecting patients to undergo bariatric surgery.

## 2. Material and Methods

### 2.1. Study Design

Study population and study design have been described previously [23]. Study subjects with type 2 diabetes or NGT were recruited from the Hvidovre Hospital Bariatric surgery program (Hvidovre, Denmark). They all met the Danish criteria for bariatric surgery: >20 years of age and a body mass index (BMI) >40 kg/m² or >35 kg/m² with comorbidities. 

NGT was defined at the time of entry into the bariatric surgery programme as fasting plasma glucose <6.1 mM and 2 h plasma glucose of <7.8 mM after a 75 g oral glucose load. Type 2 diabetes was defined as (1) treatment with ≥1 antidiabetic agent(s) and a fasting plasma glucose of  >7.0 mM on the first study day, prior to the operation; or (2) in diet- treated subjects, a confirmatory oral glucose tolerance test was performed two weeks before surgery (2 h plasma glucose of ≥11.1 mM). Exclusion criteria were treatment with thyroid hormone substitution therapies, antithyroid medication or anorectics, and incretin-based therapies or insulin within 3 months prior to the first study day. Subjects were studied approximately 3 days prior to (pre) and 1 wk, 3 mo, and 1 yr after surgery. After the entry into the bariatric surgery programme, but prior to inclusion in the present study and the operation, all study subjects had accomplished a diet-induced weight loss of 8% of their total body weight. 

 Antidiabetic medications were paused 72 h before the preoperative meal test. On each study day, preceded by a 10-hour overnight fast, anthropometric measures were retrieved, including weight (Tanita Corp., Japan), height, abdominal and hip circumference, and blood pressure (A & D Co., Ltd., Saitama, Japan). BMI (kg/m^2^) and waist-hip ratio (WHR, waist circumference/hip circumference) were calculated. The participants were given a liquid meal consisting of 200 mL of Fresubin Energy Drink (300 kcal, carbohydrate (E% 50), protein (E%15), fat (E% 35), Fresenius Kabi Deutschland, Bad Homburg, Germany). Blood samples were obtained at meal start and frequently during the meal (0, 30, 45, 60, 90, 120, 180, and 240 minutes relative to meal start). During each meal test, the participants sat in a reclined position; no strenuous activity was allowed.

### 2.2. Surgical Procedure

Surgery was performed at the Department of Gastroenterology at Hvidovre Hospital (Hvidovre, Denmark) by either of two surgeons (L. Naver or L. Hvolris, both consultants in gastrosurgery and with expertise in RYGB), using a standard laparoscopic RYGB technique [23].

### 2.3. Laboratory Analyses

Serum insulin and C-peptide levels were measured by AutoDELFIA fluoroimmunoassay (Wallac OY, Turku, Finland). HbA1c was determined using HPLC with a cation exchange column (Tosoh Bioscience, Tokyo, Japan), and plasma glucose was measured with the glucose oxidase technique (YSI model 2300 STAT Plus; Yellow Springs Instruments, Yellow Springs, OH, USA). Plasma FFA and triglyceride concentrations were determined using commercial *in vitro* enzymatic calorimetric assays (Wako Chemicals Gmbh, Neuss, Germany and Roche Diagnostics Gmbh, Mannheim, Germany, resp.). Serum YKL-40 was analysed using a commercial ELISA assay (Quidel, USA), with a measuring range of 20–300 ng/mL, and intra- and interassay coefficients of variation (CV) of 5.8% and 6.0%, respectively. Serum MCP-1 was measured using a Bio-Plex Pro Human Cytokine MCP-1 assay (Bio-Rad, USA) with a measuring range of 2.1–1820 pg/mL, assay sensitivity of 1.1 pg/mL, and intra- and interassay CV of 9% and 7%, respectively.

### 2.4. Statistical Analyses

Analyses were performed using the statistical software package SPSS 20.0 (SPSS Inc., Chicago, IL, USA). A two-sided *P* value < 0.05 was considered statistically significant. Continuous variables were presented as mean (standard deviation (SD)) or median (interquartile range (IQR)). Categorical variables were presented as numbers (%). Non-Gaussian distributed data as revealed by P-plot was logarithmically transformed using the natural logarithm prior to further parametric statistical analyses. 

Baseline characteristics were presented according to status (type 2 diabetes/NGT) and study day (pre, 1 wk, 3 mo, and 1 yr) (Table 1). Categorical data were compared using the Pearson chi-square test and continuous data were compared between groups (type 2 diabetes/NGT) by Independent-Samples *t*-test. To assess the possible effect of RYGB on baseline characteristics, values were compared over time (pre, 1 wk, 3 mo, 1 yr) between the two groups (type 2 diabetes/NGT) and within each group using repeated measures in a general linear model, with correction by Greenhouse-Geisser if Mauchly's test of sphericity proved to be significant. 

To assess the effect of the mixed meal tests on the inflammatory markers (YKL-40, MCP-1) and lipids (triglyceride, FFA), repeated measures were used in a similar way, comparing the values at 0, 30, 45, 60, 90, 120, 180, and 240 minutes relative to meal start within each group on each study day. To assess the possible effect of RYGB on the postprandial inflammatory response, the total area under the curve (T-AUC) was calculated using the trapezoidal model. Incremental AUC (I-AUC) was calculated as T-AUC minus baseline × 4 hours (median values and interquartile range (IQR) are displayed in Table 2). Wilcoxon matched-pairs signed-rank test was used for comparing I-AUC within groups, and for comparison between groups the Mann-Whitney *U*-test was used. Values missing from the mixed meal data set were calculated from previous values using the mean percentage change from the remaining study subjects. 

Age and gender adjusted linear regression analyses were performed for preoperative and 1 yr fasting values of YKL-40 and MCP-1 to investigate the possible association with the following variables: BMI, WHR, triglyceride, FFA, HbA1c, glucose, insulin, and IR. Furthermore, analyses of the possible correlation between YKL-40 and MCP-1 were made. The homeostasis model assessment of insulin resistance (HOMA-IR) was used to calculate IR (Insulin_fasting_ × glucose_fasting_/(22.5 × 6.945)).

**Figure 1:**
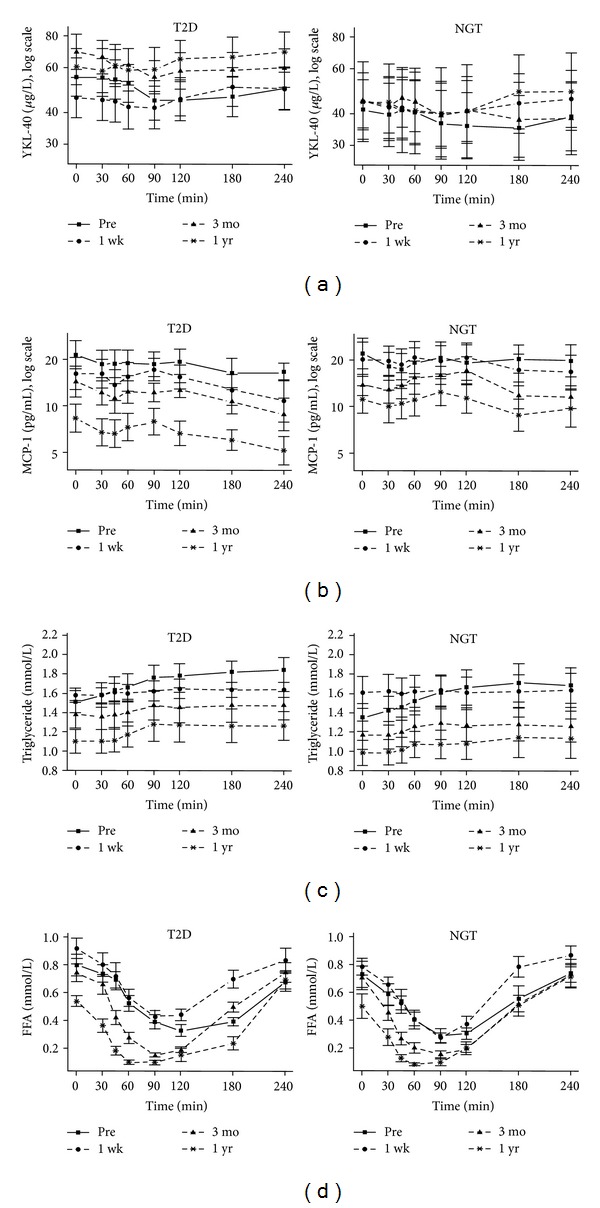
YKL-40 (a), MCP-1 (b), triglyceride (c), and free fatty acids (FFA) (d) in response to a meal in subjects with type 2 diabetes (T2D) and with normal glucose tolerance (NGT) before (pre) and 1 wk, 3 mo, and 1 yr after Roux-en-Y gastric bypass (RYGB). Data are presented as means ± standard error of mean (SEM).

Figures were created using R 2.15.2 statistical software package (R Foundation for Statistical Computing, Vienna, Austria). Values are mean ± standard error of the mean (Figure 1).

### 2.5. Ethics Statement

 All study subjects gave written informed consent, and the study was approved by the local ethics committee in accordance with the Helsinki-II declaration and by the Danish Data Protection Agency and was registered at http://www.clinicaltrials.gov/ (ClinicalTrials.gov ID NCT00810823).

## 3. Results

### 3.1. Subjects

In total, 10 obese patients with type 2 diabetes and 10 obese subjects with NGT were included in the study and examined before and after RYGB (age: type 2 diabetes, 49.9 (range 37–62) versus NGT, 42.4 (range 21–64) yrs, *P* = 0.215; gender (male/female): type 2 diabetes, 4/6 versus NGT, 3/7, *P* = 0.369). One subject with type 2 diabetes could not be studied 1 wk after RYGB because of anaemia. One subject with NGT was excluded from the 3 mo follow-up data set due to excessively high fasting insulin and C-peptide concentrations, indicating a nonfasting state.

At the time of inclusion, nine subjects with type 2 diabetes were treated with ≥1 oral antidiabetic medication, and one subject was diet treated only. The mean time from diagnosis of type 2 diabetes was 4.5 (SD 3.6) years. After surgery, none of the patients with type 2 diabetes received any antidiabetic medication. More patients in the type 2 diabetes group had comorbidities (type 2 diabetes/NGT): hypertension: 5/5; hypercholesterolemia: 3/1; atrial fibrillation : 1/0; angina pectoris: 1/0; stroke : 1/0; microalbuminuria: 2/0; arthritis: 2/0. After one year, only one subject out of 5 in the NGT group received antihypertensive medication, whereas the number was 4 out of 5 in the type 2 diabetes group. All study subjects were offered pain medication (ibumetin, 400 mg × 3/day) after surgery, that is, the 1 wk study day.

### 3.2. Clinical Characteristics, Biometrics, and Blood Pressure

Data are presented in Table 1. Both groups lost weight after RYGB; after 1 yr subjects had lost about 25% of their preoperative body weight (type 2 diabetes: 1 wk: 1.8 (SD 1.4) %; 3 mo: 13.7 (SD 3.8) %; 1 yr: 23.7 (SD 9.2) %; NGT: 1 wk: 2.4 (SD 1.4) %; 3 mo: 14.6 (SD 4.9) %; 1 yr: 24.7 (SD 8.2) %, *P* < 0.0001 for both). 

BMI and WHR did not differ between the groups prior to the operation or at any time point after RYGB (all *P* values ≥ 0.483). There was a significant decrease in BMI over time within both groups (type 2 diabetes: 42.5 (SD 5.7) kg/m^2^ −32.8 (SD 8.2) kg/m^2^; NGT: 42.5 (SD 4.6) kg/m^2^ −32.1 (SD 4.4) kg/m^2^, *P* values < 0.0001 for both), but with no overall difference between the groups (*P* = 0.738). WHR changed significantly in both groups (type 2 diabetes: *P* = 0.030; NGT: *P* = 0.017), but no differences were seen between the groups (*P* = 0.589). Systolic blood pressure did not change in the type 2 diabetes group (pre, 136 (SD 15) mmHg; 1 wk, 131 (SD 13) mmHg; 3 mo, 134 (10) mmHg; 1 yr, 138 (12) mmHg, *P* = 0.517). In the NGT group, there was a tendency towards a decrease of the systolic blood pressure; however, this was not significant (pre, 135 (SD 6) mmHg; 1 wk, 136 (SD 12) mmHg; 3 mo, 136 (SD 12) mmHg; 1 yr, 130 (SD 14) mmHg, *P* = 0.411). The diastolic blood pressure did not change in the type 2 diabetes group (pre, 83 (SD 7) mmHg; 1 wk, 79 (SD 8) mmHg; 3 mo, 81 (SD 7) mmHg; 1 yr, 83 (SD 9) mmHg, *P* = 0.512); however, in the NGT group, there was a significant decrease (pre, 81 (SD 11) mmHg; 1 wk, 75 (SD 9) mmHg; 3 mo, 78 (SD 6) mmHg; 1 yr, 73 (SD 9) mmHg, *P* = 0.010). Diastolic blood pressure differed between the groups at 1 yr (*P* = 0.036); however, no other differences of blood pressure between groups were observed (*P* values ≥ 0.214).

### 3.3. Fasting State

Data are presented in Table 1. Triglycerides decreased after RYGB in both groups (*P* values ≤ 0.017 for both), and FFA decreased in the type 2 diabetes group (*P* < 0.0001) but only showed a tendency towards a decrease in the NGT group (*P* = 0.087). There were no differences between the two groups at any study day (all *P* values ≥ 0.162). YKL-40 levels did not change significantly over time within either group (*P* values ≥ 0.120 for both), and there were no differences between the groups at any time point (all *P* values ≥ 0.156). MCP-1 levels decreased over time in both groups (*P* < 0.0001 for both), but with no significant differences between the two groups at any time-point (all *P* values ≥ 0.333).

Plasma values of glucose and HbA1c and serum values of insulin and C-peptide are presented in the Supplementary Table S1 available online at http://dx.doi.org/10.1155/2013/361781. 

### 3.4. Postprandial State

Changes in postprandial levels of inflammatory markers and lipids are shown in Figure 1, and median I-AUC YKL-40 levels are shown in Table 2. YKL-40 showed a pattern of a small postprandial decrease followed by an increase on all study days for the type 2 diabetes group (*P* values ≤ 0.021 for all) and at 1 wk for the NGT group (*P* = 0.038), whereas the changes before the operation and at 3 mo and 1 yr were nonsignificant (Figure 1(a)). I-AUC YKL-40 levels increased significantly for the type 2 diabetes group at 1 wk and 1 yr compared to baseline (pre: −31.38 (IQR −56.00; −13.72) *μ*g/L/t; 1 wk: 5.25 (IQR −10.56; 12.25) *μ*g/L/t, *P* = 0.008; 1 yr: −0.75 (IQR −12.56; 13.94) *μ*g/L/t, *P* = 0.007). There were no significant changes of I-AUC YKL-40 levels in the NGT group. I-AUC YKL-40 levels only differed significantly between the groups at 3 mo (type 2 diabetes: −27.56 (IQR −64.90; −18.00) *μ*g/L/t; NGT: −15.38 (IQR −20.31; 5.56) *μ*g/L/t, *P* = 0.022) (Table 2). 

Generally, MCP-1 levels showed a tendency towards a small postprandial decrease on most study days; however, these changes were only significant for the type 2 diabetes group at 1 wk (*P* = 0.001) and 1 yr (*P* < 0.0001) and for the NGT group at 3 mo (*P* = 0.009) (Figure 1(b)). I-AUC MCP-1 levels did not change significantly after RYGB (all *P* values ≥ 0.093), nor were there any differences between the groups on any study day (all *P* values ≥ 0.121) (Table 2). 

Postprandial levels of plasma triglyceride increased in both groups before the operation (*P* values < 0.0001 for both) and at 3 mo (*P* values 0.023–0.048), and for the type 2 diabetes group there was also a significant increase at 1 yr (*P* = 0.020) (Figure 1(c)). I-AUC-levels of triglyceride decreased significantly at 1 wk and 3 mo compared to baseline for both groups (Type 2 diabetes: pre: 0.91 (IQR 0.26; 1.58) mmol/L/t; 1 wk: 0.11 (IQR−0.18; 0.45) mmol/L/t, *P* = 0.028; 3 mo: 0.18 (IQR -0.06; 0.49) mmol/L/t, *P* = 0.013; NGT: pre: 0.92 (IQR 0.55; 1.52) mmol/L/t; 1 wk: 0.05 (IQR−0.23; 0.22) mmol/L/t, *P* = 0.007; 3 mo: 0.31 (IQR 0.10; 0.59) mmol/L/t, *P* = 0.011). No differences were observed between the groups (all *P* values ≥ 0.306) (Table 2). 

Postprandial levels of FFA showed the same pattern for both groups on all study days, a decrease followed by an increase (all *P* values < 0.0001) (Figure 1(d)). I-AUC FFA levels did not change significantly after RYGB in either of the groups (all *P* values ≥0.203), nor were there any differences between the groups (all *P* values ≥ 0.151) (Table 2). 

### 3.5. Correlations of YKL-40 and MCP-1 with BMI, WHR, Lipids, and Glucose Metabolites

 Neither YKL-40 nor MCP-1 correlated significantly with BMI, WHR, triglyceride, FFA, HbA1c, or HOMA-IR preoperatively (type 2 diabetes: *P* values = 0.063 − 0.996; NGT: *P* values = 0.063 − 0.944) or at 1 yr after the operation (type 2 diabetes: *P* values = 0.160 − 0.980; NGT: *P* values 0.081 − 0.916) in gender and age adjusted linear regression models when adjusting a.m. Bonferroni. 

## 4. Discussion

This is the first study to investigate the effects of RYGB on fasting levels as well as postprandial changes of lipids and the inflammatory markers YKL-40 and MCP-1 in obese patients with type 2 diabetes and matched NGT subject. We found that fasting levels of MCP-1 decreased after surgically induced weight loss in patients with type 2 diabetes and in participants with NGT (*P* < 0.0001 for both); however, no changes of YKL-40 fasting levels were found in either study group after RYGB. Postprandial changes of the two inflammatory markers were not, as expected, a temporary postprandial increase. On the contrary, a tendency towards decreasing postprandial MCP-1 levels in both study groups on most study days was observed, whereas postprandial changes of YKL-40 followed the same pattern on all study days in the type 2 diabetes group with a small decrease followed by an almost even-sized increase. No correlations between YKL-40 and MCP-1 or between the inflammatory markers and markers of obesity and type 2 diabetes or cardiovascular risk factors could be documented. 

Previously, it has been documented that plasma levels of YKL-40 are increased in obese patients with type 2 diabetes [12] compared to obese with NGT [12, 15]. However, we could not document a higher inflammatory burden as measured by YKL-40 and MCP-1 levels among type 2 diabetes patients compared to otherwise healthy obese subjects, perhaps explained by the severe obesity in both groups, which may have been the main contributor to the inflammation compared with type 2 diabetes per se. 

This is contrary to the findings of Catalán et al. [15], where the study population of obese patients with type 2 diabetes and obese subjects with NGT had a BMI similar to our study subjects (type 2 diabetes 47.0 ± 2.4 (SEM) kg/m^2^; NGT 44.0 ± 1.3 (SEM) kg/m^2^), but where the presurgical YKL-40 levels differed significantly between the groups. In this, however, the mean values of circulating YKL-40 in healthy lean subjects (18.9 ± 5.3 (SEM) ng/mL) and in obese subjects with NGT (approximately 24 ng/mL) were rather low compared to other studies regarding obesity [12, 14] and also compared to studies regarding YKL-40 levels in healthy subjects [29] and in the general population [30]. However, even though YKL-40 levels have been shown to increase with age, no differences between genders have been observed [31], and no normal range of YKL-40 in healthy individuals has yet been established [32]. Furthermore, it is difficult to draw direct comparison between studies, since some studies use serum values [14, 29, 30] and others use plasma values of YKL-40 [12, 15]. Also, differences due to the use of different immuno assays have to be considered [32]. The infiltration of white adipose tissue by macrophages and the following expression of inflammatory molecules by activated macrophages and adipocytes are thought to contribute to the systemic inflammation seen in obesity [4], in turn, leading to the development of IR and type 2 diabetes [33]. Thus, the “inflammatory profile” in morbidly obese type 2 diabetes patients without any major complications compared to obese with NGT may not differ at large. Another explanation could be that all of the subjects in this study lost 8% of their body weight prior to the operation and inclusion in the present study, and that any major differences between the two groups had therefore already equalized.

As it was the case in this study, fasting MCP-1 levels have previously been shown to decrease markedly after bariatric surgery in both type 2 diabetes [27] and subjects with different degrees of IR [28]. MCP-1 is known to be expressed in atherosclerotic plaques *in vivo *[34], and plasma protein levels of MCP-1 above the 75 percentile have been shown to be associated with all-cause death or myocardial infarction in a large group of patients with acute coronary syndrome [35]. MCP-1 has been shown to increase with age in a healthy elderly population, whereas no gender diffences were observed [36]. Mouse models have shown that the expression of an MCP-1 transgene in adipose tissue induces infiltration of macrophages into the adipose tissue and promotes the development of IR and that the inhibition of MCP-1 can reduce the infiltration of macrophages and the degree of IR in obese mice [18]. It is thus plausible that MCP-1 is an important link between inflammation and the development of obesity related conditions such as CVD and type 2 diabetes [37]. 

YKL-40 is considered a marker of inflammation and endothelial dysfunction, and elevated levels are found in patients with acute and stable CVD [32]. YKL-40 is secreted by a variety of cells with relations to the innate immune system [32] and is thought to facilitate the formation of lipid laden macrophages in the vascular cell wall [38], which is an important step in the formation of the atherosclerotic plaque. Previously, elevated levels of fasting serum YKL-40 in morbidly obese subjects have been documented to decrease after bariatric surgery [14]. However, we did not find a decrease in circulating YKL-40 levels following RYGB, which is in accordance with another study by Catalán et al., where a decrease of YKL-40 levels was found only after diet-induced and not surgically induced weight loss [15]. Even though we did not find an association between WHR and YKL-40 levels, these two variables have previously been shown to be closely correlated [15]. We did see a decrease of WHR; however, the volume of visceral fat may not have been reduced sufficiently to cause decreased levels of YKL-40. Alternatively, the method of RYGB or the fact that the gastrointestinal tract changes metabolically [39] can be a possible explanation for the nonaltered YKL-40 levels. 

The expression of MCP-1 is induced by various inflammatory mediators, including LPS [17]. Plasma levels of LPS and the expression of Toll-like receptor 4 (TLR4) protein in mononuclear cells have been shown to increase following a high-fat, high-carbohydrate meal (910 kcal; 41% carbohydrate, 17% protein, 42% fat) during a 3-hour period, compared to a meal rich in fruit and fibre (910 kcal; 58% carbohydrate, 15% protein, and 27% fat) [40]. Contrary to our expectations, we found that postprandial MCP-1 concentrations tended to decrease on most study days. This pattern, expressed as I-AUC MCP-1 levels, did not change after RYGB, and there were no differences between the groups. It could be speculated that the mixed meal given to our study subjects might not have contained a sufficient amount of calories (300 kcal; 50% carbohydrate, 15% protein, and 35% fat) to induce an inflammatory response. Postprandial changes of YKL-40 were small in both groups. In the type 2 diabetes group, the general pattern was a small decrease followed by an almost even sized increase; however, I-AUC YKL-40 increased significantly at 1 wk (*P* = 0.008) and 1 yr (*P* = 0.007) compared to baseline, indicating an attenuation of the postprandial decrease of YKL-40. However, the postprandial changes of the inflammatory markers are discrete and could be a chance finding.

The results from previous studies show a discrepancy, since MCP-1 levels have been found to correlate positively with both BMI and IR in one study [28], whereas another study showed no positive correlations [35]. In accordance with the latter, we did not find an association between MCP-1 and BMI.

Previously, higher YKL-40 levels have been observed with higher BMI, although a positive correlation between YKL-40 and BMI in multivariate adjusted analyses has not been documented [12, 13, 30]. We have previously speculated whether YKL-40 could induce lipolysis in adipocytes through the initiation of the mitogen-activated protein kinase (MAPK) and phosphoinositide-3-kinase (PI3K) signalling pathways and thereby influence the development of dyslipidemia [30]. In this study, however, we could not document a correlation between YKL-40 and lipids, as it has been demonstrated before [13, 16, 30, 41], and decreasing fasting levels of triglycerides and FFA were observed after RYGB, whereas fasting levels of YKL-40 did not change significantly.

YKL-40 is thought to promote the release of MCP-1 and other pro-inflammatory chemokines by alveolar macrophages from COPD patients [42]. Thus, we would have expected a positive correlation between the two markers. However, in contrast to previous findings [14, 15], we did not see this association.

One of the limitations to this study is the sample size, which could explain why we did not find a significant decrease of YKL-40 after RYGB or any significant associations of YKL-40 with MCP-1, measures of obesity, lipids, or glucose metabolism. Furthermore, the fact that we did not include lean age- and gender-matched subjects for controls makes it difficult to draw conclusions regarding the putative “normalisation” of the inflammatory state in the formerly morbid obese subjects.

## 5. Conclusions

We document decreasing fasting levels of MCP-1 over a one-year period after RYGB in obese subjects with type 2 diabetes and NGT, whereas fasting levels of YKL-40 did not change significantly. No difference in the inflammatory burden between type 2 diabetes and subjects with NGT was observed. Postprandial levels of MCP-1 tended towards a decrease; however, the postprandial changes of YKL-40 levels were less consistent. No correlations were found between YKL-40 and MCP-1 or between the inflammatory markers and obesity-related clinical features or lipid levels. 

## Supplementary Material

The fasting values of plasma glucose, serum insulin and C–peptide decreased over time in both groups (P ≤ 0.008, for all). Fasting levels of glucose differed between groups on all study days (P ≤ 0.023, for all), whereas no differences regarding serum insulin and serum C–peptide were found between the groups (P ≥ 0.171, for all). HbA1c decreased in the type 2 diabetes group (Pre: 53.0 (SD 9.8) mmol/mol; 3 mo: 41.6 (SD 9.9) mmol/mol; 1 yr: 37.7 (SD 6.8) mmol/mol; P = 0.001), whereas no significant change was observed in the NGT group (P = 0.161). The HbA1c- values differed between the groups prior to the operation (P < 0.0001) and at 3 mo (P = 0.047), but not at 1 yr (P = 0.270).

## Figures and Tables

**Table 1:** Clinical characteristics, lipids and inflammatory markers in subjects with type 2 diabetes, and normal glucose tolerance before (pre) and 1 wk, 3 mo, and 1 yr after Roux-en-Y gastric bypass.

		Pre	1 wk	3 mo	1 yr	*P* value^§^	*P* value^II^
*N* (% of total)	T2D	10 (50)	9 (47)	10 (53)	10 (50)		
	NGT	10 (50)	10 (53)	9 (47)	10 (50)		

Systolic BP, mmHg*	T2D	136 (15)	131 (13)	134 (10)	138 (12)	0.517	0.295
	NGT	135 (6)	136 (12)	136 (12)	130 (14)	0.411	
	*P* value^‡^	0.876	0.432	0.788	0.214		

Diastolic BP, mmHg*	T2D	83 (7)	79 (8)	81 (7)	83 (9)	0.512	0.117
	NGT	81 (11)	75 (9)	78 (6)	73 (9)	**0.010**	
	*P* value^‡^	0.598	0.313	0.383	0.036		

Weight, Kg*	T2D	125.9 (14.4)	124.5 (13.9)	109.0 (15.6)	96.9 (20.6)	**<0.0001**	0.578
	NGT	130.3 (10.4)	127.2 (9.6)	108.8 (7.6)	98.0 (12.1)	**<0.0001**	
	*P* value^‡^	0.441	0.628	0.972	0.886		

BMI, Kg/m^2∗^	T2D	42.5 (5.7)	41.9 (6.0)	36.8 (6.1)	32.8 (8.2)	**<0.0001**	0.738
	NGT	42.5 (4.6)	41.4 (4.2)	35.2 (2.9)	32.1 (4.4)	**<0.0001**	
	*P* value^‡^	0.977	0.830	0.483	0.797		

WHR*	T2D	1.0 (0.1)	1.1 (0.1)	1.1 (0.1)	1.0 (0.1)	**0.030**	0.589
	NGT	1.1 (0.1)	1.1 (0.1)	1.0 (0.1)	1.0 (0.1)	**0.017**	
	*P* value^‡^	0.917	0.650	0.647	0.832		

TG, mmol/L*	T2D	1.51 (0.36)	1.58 (0.22)	1.38 (0.45)	1.10 (0.39)	**0.017**	0.385
	NGT	1.35 (0.46)	1.61 (0.53)	1.17 (0.44)	0.98 (0.41)	**0.005**	
	*P* value^‡^	0.400	0.867	0.311	0.512		

FFA, mmol/L*	T2D	0.80 (0.25)	0.92 (0.22)	0.74 (0.20)	0.54 (0.12)	**<0.0001**	0.859
	NGT	0.73 (0.30)	0.78 (0.19)	0.70 (0.26)	0.50 (0.28)	0.087	
	*P* value^‡^	0.587	0.162	0.710	0.690		

YKL-40, *µ*g/L^†^	T2D	51 (37; 88)	47 (35; 71)	62 (49; 110)	59 (35; 94)	0.120	0.362
	NGT	43 (22; 99)	61 (31; 76)	44 (31; 88)	51 (27; 97)	0.930	
	*P* value^‡^	0.388	0.947	0.156	0.449		

MCP-1, pg/mL^†^	T2D	25.2 (11.3; 35.5)	20.4 (9.0; 24.4)	15.9 (8.6; 19.9)	9.7 (6.4; 11.9)	**<0.0001**	0.213
	NGT	14.9 (12.4; 42.1)	17.8 (13.7; 34.4)	13.1 (9.2; 22.9)	11.1 (6.6; 16.1)	**<0.0001**	
	*P* value^‡^	0.920	0.533	0.887	0.333		

Data are presented as *mean (SD), ^†^median (IQR), or number (%) where not specified.

^‡^ *P* value for comparison of T2D and NGT; Independent Samples *t*-test.

^§^ *P*-value for overall comparison between days within a group (T2D or NGT); repeated measures.

^II^ *P* value for overall comparison of changes between the groups (T2D and NGT); repeated measures.

BMI: body mass index; DBP: diastolic blood pressure; FFA: free fatty acids; MCP-1: monocyte chemoattractant protein-1; NGT: normal glucose tolerance; SBP: systolic blood pressure; T2D: type 2 diabetes; TG: triglyceride; WHR: waist-hip ratio.

**Table 2:** I-AUC values for postprandial concentrations of YKL-40, MCP-1, FFA, and triglycerides in subjects with type 2 diabetes and normal glucose tolerance before (pre) and 1 wk, 3 mo, and 1 yr after Roux-en-Y gastric bypass.

		Pre	1 wk	*P* value^‡^	3 mo	*P* value^‡^	1 yr	*P* value^‡^
*N* (% of total)	T2D	10 (50)	9 (47)		10 (53)		10 (50)	
	NGT	10 (50)	10 (53)		9 (47)		10 (50)	

I-AUC YKL-40, *μ*g/L/t*	T2D	−31.38 (−56.0; −13.72)	5.25 (−10.56; 12.25)	**0.008**	−27.56 (−64.90; −18.00)	0.866	−0.75 (−12.56;13.94)	**0.007**
	NGT	−18.44 (−31.69; −6.41)	−0.19 (−16.53; 6.60)	0.386	−15.38 (−20.31; 5.56)	0.260	−2.75 (−13.35; −17.78)	0.114
	*P* value^†^	0.174	0.414		**0.022**		0.821	

I-AUC MCP1, pg/mL/t*	T2D	−14.62 (−36.38; −0.19)	−8.81 (−16.58; −2.33)	0.139	−8.80 (−27.02; 0.67)	0.721	−7.65 (−17.14; −1.39)	0.114
	NGT	−7.78 (−21.28; −1.42)	−7.79 (−14.02; −1.63)	0.445	−1.37 (−11.52; 21.01)	0.441	−3.32 (−5.69; 2.65)	0.093
	*P* value^†^	0.705	0.935		0.121		0.226	

I-AUC FFA, mmol/L/t*	T2D	−1.14 (−1.84; −0.61)	−0.82 (−1.66; −0.64)	0.859	−1.16 (−1.52; −0.94)	0.878	−1.16 (−1.38; −0.64)	0.508
	NGT	−0.69 (−1.71; 0.49)	−0.68 (−1.19; −0.37)	0.333	−1.36 (−1.75; −0.78)	0.441	−0.77 (−1.24; 0.03)	0.203
	*P* value^†^	0.450	0.191		0.806		0.151	

I-AUC TG, mmol/L/t*	T2D	0.91 (0.26; 1.58)	0.11 (−0.18; 0.45)	**0.028**	0.18 (−0.06; 0.49)	**0.013**	0.46 (0.03; 0.70)	0.059
	NGT	0.92 (0.55; 1.52)	0.05 (−0.23; 0.22)	**0.007**	0.31 (0.10; 0.59)	**0.011**	0.30 (0.06; 0.60)	0.074
	*P* value^†^	0.762	0.306		0.414		0.677	

*Median (IQR).

^†^ *P* value: comparison between groups (T2D/NGT). Mann-Whitney *U-*test.

^‡^ *P* value: compared to baseline (pre). Wilcoxon matched-pairs signed-rank test.

I-AUC: incremental area under the curve; MCP-1: monocyte chemoattractant protein-1; FFA: free fatty acids; TG: triglycerides; T2D: type 2 diabetes; NGT: normal glucose tolerance.
