# Supplementary material for: Effects of Roux-en-Y Gastric Bypass on Fasting and Postprandial Levels of the Inflammatory Markers YKL-40 and MCP-1 in Patients with Type 2 Diabetes and Glucose Tolerant Subjects
Source: J Obes. 2013 Nov 3;2013:361781. doi: 10.1155/2013/361781 (PMC3835778; doi:10.1155/2013/361781)
Supplement: Supplementary file 1 — The fasting values of plasma glucose, serum insulin and C–peptide decreased over time in both groups (P ≤ 0.008, for all). Fasting levels of glucose differed between groups on all study days (P ≤ 0.023, for all), whereas no differences regarding serum insulin and serum C–peptide were found between the groups (P ≥ 0.171, for all). HbA1c decreased in the type 2 diabetes group (Pre: 53.0 (SD 9.8) mmol/mol; 3 mo: 41.6 (SD 9.9) mmol/mol; 1 yr: 37.7 (SD 6.8) mmol/mol; P = 0.001), whereas no significant change was observed in the NGT group (P = 0.161). The HbA1c- values differed between the groups prior to the operation (P < 0.0001) and at 3 mo (P = 0.047), but not at 1 yr (P = 0.270). [file 361781.f1.doc]

Supplementary table S1. Fasting levels of plasma glucose and HbA1c, serum insulin and C-peptide in subjects with type 2 diabetes and normal glucose tolerance before (pre), and 1 wk, 3 mo and 1 yr after Roux-en-Y gastric bypass

|  |  | Pre | 1wk | 3mo | 12mo | p-value§ | p-valueІІ |
| --- | --- | --- | --- | --- | --- | --- | --- |
| N  (% of total) | T2D | 10 (50) | 9 (47) | 10 (53) | 10 (50) |  |  |
|  | NGT | 10 (50) | 10 (53) | 9 (47) | 10 (50) |  |  |
| Glucose,  mmol/l* | T2D | 8.9 (2.5) | 6.7 (1.3) | 6.6 (1.7) | 6.2 (1.8) | **<0.0001** | **0.007** |
|  | NGT | 5.5 (0.7) | 5.0 (0.7) | 4.9 (0.4) | 5.0 (0.3) | **0.008** |  |
|  | p-value‡ | **<0.0001** | **0.001** | **0.005** | **0.023** |  |  |
| HbA1c,  mmol/mol* | T2D | 53.0 (9.8) |  | 41.6 (9.9) | 37.7 (6.8) | **0.001** | **0.006** |
|  | NGT | 36.5 (4.5) |  | 34.3 (2.9) | 34.8 (3.7) | 0.161 |  |
|  | p-value‡ | **<0.0001** |  | **0.047** | 0.270 |  |  |
| Insulin,  pmol/l† | T2D | 104  (49; 167) | 54  (42; 72) | 39  (23; 53) | 24  (21; 67) | **<0.0001** | 0.395 |
|  | NGT | 71  (58; 78) | 45  (37; 58) | 40  (31; 50) | 34  (21; 52) | **0.001** |  |
|  | p-value‡ | 0.228 | 0.554 | 0.882 | 0.891 |  |  |
| C-peptide, pmol/l† | T2D | 1298  (886; 1786) | 1017  (580; 1146) | 744  (551; 996) | 532  (456; 875) | **<0.0001** | 0.284 |
|  | NGT | 1062  (980; 1178) | 726  (610; 944) | 786.0  (662.0; 875.5) | 571  (430; 704) | **<0.0001** |  |
|  | p-value‡ | 0.171 | 0.632 | 0.913 | 0.681 |  |  |

Data are presented as *mean (SD), †median (IQR) or number (%) where not specified.

‡p-value for comparison of T2D and NGT; Independent samples T-test .

§p-value for overall comparison between days within a group (T2D or NGT); repeated measures.

ІІp-value for overall comparison of changes between the groups (T2D and NGT); repeated measures.

Abbreviations: HbA1c, glycated hemoglobin; T2D, type 2 diabetes; NGT, normal glucose tolerance.
